# Peripheral Intravenous Catheters in Hospitalized Surgical Patients: A US Real-World Data Analysis

**DOI:** 10.36469/001c.156489

**Published:** 2026-04-10

**Authors:** Rena Moon, Julie Gayle, Stephanie Pitts, Joy David, Kristin Jacobs, Ning Rosenthal

**Affiliations:** 1 Premier Applied Sciences, Premier Inc., Charlotte, North Carolina; 2 B. Braun Medical Inc., Bethlehem, Pennsylvania

**Keywords:** peripheral intravenous catheter, complication, cost, readmission, bloodstream infection

## Abstract

**Background:**

Limited evidence exists to evaluate the clinical and economic burden of treating hospitalized surgical patients experiencing peripheral intravenous catheter (PIVC)–associated complications.

**Objective:**

To estimate the prevalence of PIVC-associated complications and compare the healthcare resource use and cost between surgical inpatients with and without a PIVC-associated complication.

**Methods:**

This retrospective cohort study used a large, geographically diverse, hospital-based US database (Premier Healthcare Database). Hospitalized adult (≥18 years) and pediatric (<18 years) patients undergoing a surgery between January 1, 2019, and December 31, 2023, without the use of central line access device were included.

**Results:**

The analysis included 6 992 120 adult and 159 256 pediatric patients. In the adult cohort, the prevalence of PIVC-associated complications was 0.7%. Patients with complications were older (mean, 64.2 vs 55.2 years) and more likely to be men (53.1% vs 36.6%) than those without complications (both P < .01). Patients with complications were 46% more likely to be readmitted for any reason (odds ratio [OR], 1.46; 95% confidence interval [CI], 1.42-1.50), had longer length of stay (LOS) by 5.52 days, and incurred higher costs by $19 074 than patients without complications, after adjusting for covariates (all *P* < .01). In the pediatric cohort, the prevalence of PIVC-associated complications was 0.5%. Patients with complications were younger (mean, 8.6 vs 9.6 years) and more likely to be Black (19.9% vs 15.6%) than those without complications (both *P* < .01). Patients with complications were 115% more likely to be readmitted for any reason within 30 days after discharge (OR, 2.15; 95% CI, 1.73-2.67), had longer LOS by 4.50 days, and incurred higher costs by $16 052 than patients without complications, after adjusting for covariates (all *P* < .01).

**Discussion:**

While the overall prevalence of PIVC-associated complications is around 1%, this still amounts to a significant number of patients as most patients undergoing an inpatient surgical procedure would have a PIVC placed. The study results call for stakeholders to establish a process for decreasing complications related to PIVCs.

**Conclusions:**

In both adult and pediatric cohorts, patients with PIVC-associated complications had significantly higher total hospitalization cost, LOS, and 30-day readmission risks than those without complications.

## BACKGROUND

Over 170 million peripheral intravenous catheters (PIVCs) are inserted every year and complications with their use are not uncommon.^1,2^ Complications associated with PIVCs include but are not limited to catheter-related bloodstream infection (BSI), catheter occlusion, phlebitis, and infiltration.^1,3^ Among these, the most serious complication associated with PIVC is BSI.^1,4^ Because the protective barrier effect of the skin is breached when a PIVC is inserted, bacteria can enter the bloodstream or cause local infections (eg, cellulitis, soft tissue infection).^1,4,5^ Therefore, common pathogens associated with PIVC-associated BSI are normal skin flora or those commonly found in hospital settings (eg, coagulase-negative *Staphylococci* and *Staphylococcus aureus).*^6^ A recent study of 110 PIVC tips in Portugal reported that 30% were contaminated with pathogens, of which the majority was *Staphylococcus* species with high pathogenicity and resistance to antibiotics.^7^ The prevalence of PIVC-associated BSI in the United States is estimated to be around 0.2%.^8^ However, this percentage may be underreported because PIVCs are not consistently tracked for BSI in national surveillance systems.^9,10^ Not only can catheter-related BSI significantly prolong hospital stay and increase healthcare cost, but it can also put patients at risk of developing sepsis, multi-organ failure, and mortality.^1,11,12^

Other complications such as occlusion and phlebitis can lead to failure and premature removal of catheters.^13^ Catheter failure may require catheter replacement, increasing healthcare resource use and cost (eg, new catheters, nursing time) and delaying time-sensitive treatment for patients such as chemotherapy or antibiotic administration.^13,14^ Repeated PIVC failure can also lead to patients needing central venous access devices that are already associated with higher risk of complications and cost.^15^

Several national organizations provide guidelines for proper PIVC insertion and for prevention and management of catheter-related infections,^16–18^ yet nurses typically receive little instruction in PIVC insertion and only about 57% of nursing students receive PIVC training.^19,20^ In addition, executive-level hospital staff may be unaware of complications due to PIVC and the need for additional nurse training,^21^ and they may not consider PIVC-associated complications to be an area for improvement.^22^

This study sought to examine the prevalence of PIVC-associated complications in a large national sample of surgical patients and assess their burden on healthcare by comparing hospitalization cost, length of stay (LOS), and 30-day readmission risk between patients with complications and those without.

## METHODS

### Data Source

This study used data from the Premier Healthcare Database (PHD). The PHD is an all-payer hospital administrative database containing more than 1.5 billion inpatient and outpatient encounters from over 1300 geographically diverse hospitals in the United States. The standard hospital discharge files include patient demographics, disease states, and time-stamped log of billed services, including medications, laboratory, diagnostics, and therapeutic services at the patient-level, and hospital characteristics including geographic location, urbanicity of served population, teaching status, and bed capacity. All discharge data are statistically deidentified and compliant with the Health Insurance Portability and Accountability Act (HIPAA). Based on 45 CFR § 46, the study was exempted from institutional review board approval. We did not pursue informed consent of study participants due to the nature of de-identified data. The study followed the Strengthening the Reporting of Observational Studies in Epidemiology (STROBE)^23^ reporting guideline.

### Study Design and Population

This was a retrospective observational study of adult (age ≥18 years) and pediatric (age <18 years) surgical inpatients discharged between January 1, 2019, and December 31, 2023. Surgical inpatients were defined based on the presence of a Medicare Severity Diagnostic Related Group surgical code. Patients with peripherally inserted central catheter (PICC) or a central venous catheter (CVC) during the same hospitalization were excluded. Patients with multiple surgical inpatient discharges and those from hospitals without continuous data submission during the 180-day look-back and 30-day follow-up periods were excluded as well.

### Study Measures

The primary outcome was PIVC-associated complications, which included BSI, extravasation including infiltration, upper extremity cellulitis, upper extremity phlebitis, acute embolism or thrombosis, and vascular complications following infusion, transfusion, or therapeutic injection. PIVC-associated complications were identified using *International Classification of Diseases, Tenth Revision, Clinical Modification* (ICD-10-CM) (**Supplementary Table S1**). If a patient had any of these complications, they were considered as having a PIVC-associated complication. Secondary outcomes included total hospital LOS, total hospitalization cost (all cost incurred in the hospital during the hospitalization), and 30-day risk of all-cause readmission (percentage of patients experiencing any hospitalization during the 30-day follow-up period).

Baseline patient characteristics including age, sex, race, ethnicity, insurance payer, and hospital characteristics including urbanicity of served population, teaching status, geographic region, and hospital size were provided by the hospitals. Charlson-Deyo comorbidities for adults and Feudtner Complex Chronic Conditions for pediatric patients were identified during hospitalization and during any visit to the same hospital within 180 days prior to the hospitalization using ICD-10-CM and *International Classification of Diseases, Tenth Revision, Procedural Coding System* (ICD-10-PCS) codes.^24–26^ Charlson-Deyo Comorbidity Index score was calculated using a previously validated method.^24,27^ Cost variables were set to 96% winsorization (ie, all observations were set to have a maximum value at the 98th percentile and a minimum value at the 2nd percentile). All costs were adjusted to 2023 US dollars based on the Consumer Price Index for hospital services.^28,29^

### Statistical Analysis

Descriptive statistics were used to present baseline patient and hospital characteristics of hospitalized surgical patients and their outcomes. All analyses for adult and pediatric cohorts were conducted separately. Continuous variables were reported as mean (standard deviation [SD]) or median (first quartile, third quartile), and categorical variables were reported as counts and percentages. For statistical difference between two groups, the Student’s *t*-test or Mann-Whitney test was used for continuous variables, as indicated, and Pearson’s χ^2^ test for categorical variables.

For the primary outcome, we assessed the prevalence of PIVC-associated complications overall and for each category (BSI, extravasation including infiltration, upper extremity cellulitis, upper extremity phlebitis, acute embolism or thrombosis, and vascular complications following infusion, transfusion, and therapeutic injection). For the secondary outcomes, we stratified each cohort by PIVC-associated complication status (with complication vs without complication) and then compared the differences in LOS, total hospitalization cost, and risk of all-cause readmissions within 30 days.

Adjusted analyses to assess independent association between PIVC-associated complications and secondary outcomes were conducted using multivariable linear regression models for continuous variables (total hospitalization cost and LOS) and multivariable logistic regression models for binary variable (risk of readmission within 30 days). A priori covariates for the adult cohort were patient demographics (sex, race, ethnicity, age, insurance payer), hospital characteristics (urbanicity of served population, hospital size, teaching status, geographic region), and clinical characteristics (surgical site infection, Charlson-Deyo comorbidities). A priori covariates for the pediatric cohort included patient demographics (sex, race, ethnicity, age, insurance payer), hospital characteristics (urbanicity of served population, hospital size, teaching status, geographic region), and clinical characteristics (surgical site infection, Feudtner Complex Chronic Conditions). The final list of covariates was selected using backward selection, and only those with statistical significance at the .05 level were included in the final regression models.

All analyses were performed using R version 4.1.3 (R Foundation for Statistical Computing).

## RESULTS

We identified 7 151 376 hospitalized surgical patients meeting the study selection criteria (**Figure S1**). Of these, 6 992 120 (97.8%) patients were adults and 159 256 (2.2%) patients were children.

In the adult cohort, patients with PIVC-associated complications were older (mean age, 64.2 vs 55.2 years) and more likely to be men (53.1% vs 36.6%) and have Medicare (58.1% vs 40.5%) than those without complications (all *P* < .01). Patients with PIVC-associated complications were more likely to be treated in a large (43.8% vs 38.4%), teaching (61.1% vs 56.1%), and urban (92.4% vs 91.5%) hospital, and less likely to be discharged to home/home health (47.2% vs 84.6%) relative to patients without complications (all *P* < .01) (**Table 1** and **Supplementary Table S2A**). Compared with patients without complications, those with PIVC-associated complications were more likely to have renal disease (49.2% vs 15.9%), chronic pulmonary disease (26.8% vs 16.6%), congestive heart failure (25.3% vs 9.4%), diabetes with chronic complication (21.7% vs 10.2%), and malignancy (17.4% vs 9.0%) (all *P* < .01).

**Table 1.** Patient Demographics, Clinical Characteristics, and Hospital Characteristics of Adult and Pediatric Surgical Inpatients with PIVC-Associated Complications

**Characteristics**	**Overall**	**Patients with PIVC-Associated Complications**	**Patients without PIVC-Associated Complications**	***P***
**Adult cohort**	**N = 6 992 120**	**N = 51 294**	**N = 6 940 826**	
Mean age (SD), y	55.3 (20)	64.2 (17)	55.2 (20)	<.0001
Gender, n (%)				
Male	2 569 108 (36.8)	27 239 (53.1)	2 541 869 (36.6)	<.0001
Female	4 421 303 (63.2)	24 046 (46.9)	4 397 257 (63.4)	
Race, n (%)				
White	5 096 804 (72.9)	35 554 (69.3)	5 061 250 (72.9)	<.0001
Black	904 883 (12.9)	8241 (16.1)	896 642 (12.9)	
Other/unknown	990 433 (14.2)	7499 (14.6)	982 934 (14.2)	
Ethnicity, n (%)				
Hispanic	825 112 (11.8)	5525 (10.8)	819 587 (11.8)	<.0001
Non-Hispanic	5 325 717 (76.2)	38 597 (75.2)	5 287 120 (76.2)	
Unknown	841 291 (12.0)	7172 (14.0)	834 119 (12.0)	
Health insurance type, n (%)				
Medicare	2 844 190 (40.7)	29 814 (58.1)	2 814 376 (40.5)	<.0001
Medicaid	1 246 771 (17.8)	8023 (15.6)	1 238 748 (17.8)	
Private insurance	2 390 314 (34.2)	10 205 (19.9)	2 380 109 (34.3)	
Uninsured	226 795 (3.2)	1531 (3.0)	225 264 (3.2)	
Other/unknown	284 050 (4.1)	1721 (3.4)	282 329 (4.1)	
Charlson-Deyo comorbidities, n (%)				
Myocardial infarction	359 453 (5.1)	5745 (11.2)	353 708 (5.1)	<.0001
Congestive heart failure	663 037 (9.5)	12 985 (25.3)	650 052 (9.4)	<.0001
Peripheral vascular disease	299 420 (4.3)	4793 (9.3)	294 627 (4.2)	<.0001
Cerebrovascular disease	324 590 (4.6)	6626 (12.9)	317 964 (4.6)	<.0001
Dementia	241 240 (3.5)	4598 (9.0)	236 642 (3.4)	<.0001
Chronic pulmonary disease	1 165 700 (16.7)	13 748 (26.8)	1 151 952 (16.6)	<.0001
Rheumatic disease	143 958 (2.1)	1478 (2.9)	142 480 (2.1)	<.0001
Peptic ulcer disease	50 877 (0.7)	1881 (3.7)	48 996 (0.7)	<.0001
Mild liver disease	202 156 (2.9)	2467 (4.8)	199 689 (2.9)	<.0001
Diabetes without chronic complication	796 311 (11.4)	7089 (13.8)	789 222 (11.4)	<.0001
Diabetes with chronic complication	719 147 (10.3)	11 134 (21.7)	708 013 (10.2)	<.0001
Hemiplegia or paraplegia	94 926 (1.4)	3006 (5.9)	91 920 (1.3)	<.0001
Renal disease	1 128 249 (16.1)	25 244 (49.2)	1 103 005 (15.9)	<.0001
Moderate/severe liver disease	73 537 (1.1)	2894 (5.6)	70 643 (1.0)	<.0001
Any malignancy	637 016 (9.1)	8923 (17.4)	628 093 (9.0)	<.0001
Metastatic solid tumor	199 547 (2.9)	4054 (7.9)	195 493 (2.8)	<.0001
HIV disease	9653 (0.1)	219 (0.4)	9434 (0.1)	<.0001
CCI score category				
0	3 283 879 (47.0)	7649 (14.9)	3 276 230 (47.2)	<.0001
1-2	2 097 890 (30.0)	12 838 (25.0)	2 085 052 (30.0)	
>2	1 610 351 (23.0)	30 807 (60.1)	1 579 544 (22.8)	
Mean CCI score (SD)	1.56 (2)	3.75 (3)	1.54 (2)	<.0001
**Pediatric cohort**	**N=159 256**	**N = 816**	**N = 158 440**	
Mean age (SD), y	9.5 (6)	8.6 (6)	9.6 (6)	<.0001
Gender, n (%)				
Male	85 610 (53.8)	433 (53.1)	85 177 (53.8)	0.6867
Female	73 617 (46.2)	383 (46.9)	73 234 (46.2)	
Race, n (%)				
White	95 889 (60.2)	485 (59.4)	95 404 (60.2)	0.0011
Black	24 862 (15.6)	162 (19.9)	24 700 (15.6)	
Other	38 505 (24.2)	169 (20.7)	38 336 (24.2)	
Ethnicity, n (%)				
Hispanic	38 721 (24.3)	167 (20.5)	38 554 (24.3)	<.0001
Non-Hispanic	101 121 (63.5)	401 (49.1)	100 720 (63.6)	
Unknown	19 414 (12.2)	248 (30.4)	19 166 (12.1)	
Health insurance type, n (%)				
Medicare	235 (0.1)	3 (0.4)	232 (0.1)	<.0001
Medicaid	88 446 (55.5)	497 (60.9)	87 949 (55.5)	
Private insurance	60 161 (37.8)	262 (32.1)	59 899 (37.8)	
Uninsured	3791 (2.4)	35 (4.3)	3756 (2.4)	
Other/unknown	6623 (4.2)	19 (2.3)	6604 (4.2)	
Feudtner Complex Chronic Conditions, n (%)				
Neurologic and neuromuscular	14 874 (9.3)	119 (14.6)	14 755 (9.3)	<.0001
Cardiovascular	13 334 (8.4)	163 (20.0)	13 171 (8.3)	<.0001
Respiratory	4870 (3.1)	88 (10.8)	4782 (3.0)	<.0001
Renal and urologic	5664 (3.6)	52 (6.4)	5612 (3.5)	<.0001
Gastrointestinal	12 930 (8.1)	175 (21.4)	12 755 (8.1)	<.0001
Hematologic or immunologic	3781 (2.4)	73 (8.9)	3708 (2.3)	<.0001
Metabolic	5138 (3.2)	106 (13.0)	5032 (3.2)	<.0001
Other congenital or genetic defect	0 (0.0)	0 (0.0)	0 (0.0)	NA
Premature and neonatal	1817 (1.1)	51 (6.2)	1766 (1.1)	<.0001
Malignancy	4670 (2.9)	45 (5.5)	4625 (2.9)	<.0001
Technology dependence	14 308 (9.0)	185 (22.7)	14 123 (8.9)	<.0001
Solid organ/bone marrow transplantation	609 (0.4)	13 (1.6)	596 (0.4)	<.0001

Abbreviations: CCI, Charlson-Deyo Comorbidity Index; NA, not applicable; PIVC, peripheral intravenous catheter.

In the pediatric cohort, patients with PIVC-associated complications were younger (mean age, 8.6 vs 9.6 years), more likely to be Black (19.9% vs 15.6%) and have Medicaid (60.9% vs 55.5%) than those without complications (all *P* < .01). Patients with complications were treated more often in teaching (85.3% vs 76.8%) and urban (98.3% vs 96.4%) hospitals and were less likely to be discharged to home/home health (86.5% vs 97.6%) (all *P *< .01) (**Table 1** and **Supplementary Table S2B**).

Patients with PIVC-associated complications were more likely to have technology dependence (22.7% vs 8.9%), gastrointestinal disorders (21.4% vs 8.1%), cardiovascular diseases (20.0% vs 8.3%), and neurologic or neuromuscular conditions (14.6% vs 9.3%) than their counterparts without complications (all *P* < .01).

### PIVC-Associated Complications

The prevalence of having any PIVC-associated complication was 0.70% among adult patients and 0.50% among pediatric patients (**Table 2**). BSI accounted for the majority of the complications in both adult (71.1%) and pediatric populations (71.8%). The prevalences of extravasation including infiltration, upper extremity cellulitis, upper extremity phlebitis, acute embolism/thrombosis, and vascular complications ranged from 0.02% to 0.12% in adults and from 0.01% to 0.06% in pediatric patients (**Table 2**).

**Table 2.** PIVC-Associated Complications and Healthcare Resource Use and Cost of Surgical Inpatients

**Outcomes**	**Overall**	**Patients with PIVC-Associated Complications**	**Patients without PIVC-Associated Complications**	***P***
**Adult cohort**	**N = 6 992 120**	**N = 51 294**	**N = 6 940 826**	
Prevalences of PIVC-associated complications, n (%)				
Any	51 294 (0.70)			
BSI	36 495 (0.52)			
Extravasation including infiltration	2492 (0.04)			
Upper extremity cellulitis	1666 (0.02)			
Upper extremity phlebitis	4477 (0.06)			
Acute embolism/thrombosis	8240 (0.12)			
Vascular complications	1054 (0.02)			
Healthcare resource use				
Total LOS during hospitalization (days)				
Mean (SD)	4.06 (3.77)	12.50 (6.65)	3.99 (3.67)	<.0001
Median (Q1-Q3)	3 (2-5)	11 (7-19)	3 (2-5)	
Readmissions and outpatient visits during 30-day follow-up, n (%)				
All-cause readmissions	426 286 (6.1)	6186 (13.5)	420 100 (6.1)	<.0001
All-cause outpatient visits	1 322 760 (19.0)	10 411 (22.8)	1 312 349 (19.0)	<.0001
BSI-related readmissions	67 771 (1.0)	1777 (3.9)	65 994 (1.0)	<.0001
BSI-related outpatient visits	130 (0.0)	1 (0.0)	129 (0.0)	0.616
Healthcare cost				
Total hospitalization cost (2023 US $)				
Mean (SD)	20 695 (16 764)	48 624 (29 917)	20 489 (16 452)	<.0001
Median (Q1-Q3)	15 645 (10 259-24 760)	40 130 (24 087-69 867)	15 574 (10 227-24 551)	
Total cost for all-cause readmission during 30-day follow-up (2023 US $)				
Mean (SD)	18 205 (18 796)	26 017 (25 814)	18 117 (18 683)	<.0001
Median (Q1-Q3)	12 164 (6875-22 044)	16 545 (8809-32 979)	12 128 (6859-21 944)	
Total cost for all-cause outpatient visits during 30-day follow-up (2023 US $)				
Mean (SD)	1813 (3333)	2094 (3478)	1811 (3332)	<.0001
Median (Q1-Q3)	560 (166-1713)	725 (212-2311)	559 (166-1708)	
**Pediatric cohort**	**N=159 256**	**N = 816**	**N = 158 440**	
Prevalences of PIVC-associated complications, n (%)				
Any	816 (0.50)			
BSI	586 (0.37)			
Extravasation including infiltration	95 (0.06)			
Upper extremity cellulitis	31 (0.02)			
Upper extremity phlebitis	56 (0.04)			
Acute embolism/thrombosis	80 (0.05)			
Vascular complications	8 (0.01)			
Healthcare resource use				
Total LOS during hospitalization (days)				
Mean (SD)	3.57 (3.74)	10.41 (7.66)	3.54 (3.68)	<.0001
Median (Q1-Q3)	2 (1-4)	7 (4-19)	2 (1-4)	
Readmissions and outpatient visits during 30-day follow-up, n (%)				
All-cause readmissions	7870 (5.0)	105 (13.2)	7765 (4.9)	<.0001
All-cause outpatient visits	30 486 (19.2)	216 (27.2)	30 270 (19.1)	<.0001
BSI-related readmissions	477 (0.3)	14 (1.8)	463 (0.3)	<.0001
BSI-related outpatient visits	1 (0.0)	0 (0.0)	1 (0.0)	0.99
Healthcare cost				
Total hospitalization cost (2023 US $)				
Mean (SD)	20 542 (20 473)	46 610 (36 758)	20 408 (20 270)	<.0001
Median (Q1-Q3)	13 191 (8483-23 055)	30 260 (13 826-90 624)	13 160 (8468-22 928)	
Total cost for all-cause readmission during 30-day follow-up (2023 US $)				
Mean (SD)	15 922 (19 812)	36 415 (34 753)	15 785 (19 604)	<.0001
Median (Q1-Q3)	9429 (6192-15 811)	21 852 (11 763-50 354)	9391 (6181-15 713)	
Total cost for all-cause outpatient visits during 30-day follow-up (2023 US $)				
Mean (SD)	1561 (2986)	2237 (3876)	1556 (2978)	0.0112
Median (Q1-Q3)	451 (166-1354)	769 (323-2062)	450 (166-1351)	

Abbreviations: BSI, bloodstream infection; CCI, Charlson-Deyo Comorbidity Index; LOS, length of stay; NA, not applicable; PIVC, peripheral intravenous catheter.

### Readmissions Within 30 Days

In the adult cohort, a total of 426 286 patients (6.1%) were readmitted within 30 days after hospitalization (**Table 2**). A higher proportion of patients with PIVC-associated complications were readmitted for any reason (13.5% vs 6.1%, *P* < .01) compared with those without complications. Patients with PIVC-associated complications were also more likely to have all-cause outpatient visits (22.8% vs 19.0%, *P* < .01) and BSI-related readmissions (3.9% vs 1.0%, *P* < .01) within 30 days than those without complications. After adjusting for covariates, patients with PIVC-associated complications were still 46% more likely to be readmitted for any reason than those without complications (adjusted odds ratio [aOR], 1.46; 95% confidence interval [CI], 1.42-1.50) (**Table 3**).

**Table 3.** 30-Day All-Cause Readmission Outcome Associated with PIVC-Associated Complications

	**Unadjusted OR (95% CI)**		**Adjusted OR (95% CI)**	
**With PIVC-Associated Complications**	**Without PIVC-Associated Complications**	**With PIVC-Associated Complications**	**Without PIVC-Associated Complications**	
30-day all-cause readmission				
Adult patients^a^	2.13 (2.07-2.19)	1.00 (-)	1.46 (1.42-1.50)	1.00 (-)
Pediatric patients^b^	2.67 (2.33-3.52)	1.00 (-)	2.15 (1.73-2.67)	1.00 (-)

Abbreviations: CI, confidence interval; OR, odds ratio; PIVC, peripheral intravenous catheter.

^a^Adjusted for sex, race, age group, ethnicity, insurance, urbanicity of served population, hospital size, hospital teaching status, geographic region, surgical site infection, diabetes, congestive heart failure, chronic pulmonary disease, renal disease, malignancy, and Charlson Comorbidity Index score.

^b^Adjusted for sex, age group, ethnicity, insurance, urbanicity of served population, hospital size, hospital teaching status, geo-graphic region, surgical site infection, and complex chronic conditions (neurologic and neuromuscular, cardiovascular, gastrointestinal, and technology dependence).

Among the pediatric cohort, a total of 7870 patients (5.0%) were readmitted within 30 days following the hospitalization (**Table 2**). A higher proportion of those patients with PIVC-associated complications were readmitted for any reason (13.2% vs 4.9%, *P* < .01) compared to those without complications. Patients with PIVC-associated complications had higher rates of all-cause outpatient visits (27.2% vs 19.1%, P < .01) and BSI-related readmissions (1.8% vs 0.3%, *P* < .01) within 30 days than those without complications. After adjusting for covariates, patients with PIVC-associated complications had a 115% higher risk of readmission for any reason than those without complications (aOR, 2.15; 95% CI, 1.73-2.67) (**Table 3**).

### Hospital Length of Stay

In the adult cohort, the mean LOS was longer for patients with PIVC-associated complications than for those without (12.50 vs 3.99 days, *P* < .01) (**Tables 2 and 4**). After adjusting for covariates, the difference attenuated, but patients with PIVC-associated complications still had longer LOS by 5.52 days than patients without complications.

**Table 4.** Length of Stay and Total Hospitalization Cost Outcomes During Hospitalization Associated with PIVC-Associated Complications

	**N**	**Unadjusted Means (SD)**		**Adjusted Means (SD)**		**Difference (SD)**	**Ratio (95% CI)**
**With PIVC-Associated Complications**	**Without PIVC-Associated Complications**	**With PIVC-Associated Complications**	**Without PIVC-Associated Complications**				
Length of hospital stay (days)							
Adult patients^a^	6 992 120	12.50 (6.65)	3.99 (3.67)	9.53 (3.13)	4.00 (1.32)	5.52 (1.82)	2.38 (2.37-2.39)
Pediatric patients^b^	159 256	10.41 (7.66)	3.54 (3.68)	8.04 (3.23)	3.55 (1.43)	4.50 (1.81)	2.27 (2.17-2.37)
Total hospitalization cost, US $							
Adult patients^a^	6 959 775	48 624 (29 917)	20 489 (16 452)	39 626 (12 546)	20 551 (6507)	19 074 (6040)	1.93 (1.92-1.94)
Pediatric patients^b^	158 867	46 610 (36 758)	20 407 (20 270)	36 659 (17 507)	20 607 (9841)	16 052 (7666)	1.78 (1.67-1.89)

Abbreviations: CI, confidence interval; PIVC, peripheral intravenous catheter.

^a^Adjusted for sex, race, age group, ethnicity, insurance, urbanicity of served population, hospital size, hospital teaching status, geographic region, surgical site infection, diabetes, congestive heart failure, chronic pulmonary disease, renal disease, malignancy, and Charlson Comorbidity Index score.

^b^Adjusted for race, age group, ethnicity, insurance, urbanicity of served population, hospital size, hospital teaching status, geographic region, surgical site infection, and complex chronic conditions (neurologic and neuromuscular, cardiovascular, gastrointestinal, and technology dependence).

^c^Adjusted for sex, race, age group, ethnicity, insurance, urbanicity of served population, hospital size, hospital teaching status, geographic region, surgical site infection, diabetes, congestive heart failure, chronic pulmonary disease, renal disease, malignancy, and Charlson Comorbidity Index score.

^d^Adjusted for sex, race, age group, ethnicity, insurance, urbanicity of served population, hospital size, hospital teaching status, geographic region, surgical site infection, and complex chronic conditions (neurologic and neuromuscular, cardiovascular, gastrointestinal, and technology dependence).

Similarly in the pediatric cohort, the mean LOS was longer for patients with PIVC-associated complications compared with those without (10.41 vs 3.54 days, *P* < .01) (**Tables 2 and 4**). After adjusting for covariates, patients with PIVC-associated complications still had longer LOS by 4.50 days than those without complications.

### Total Hospitalization Cost

**Figure 1.**
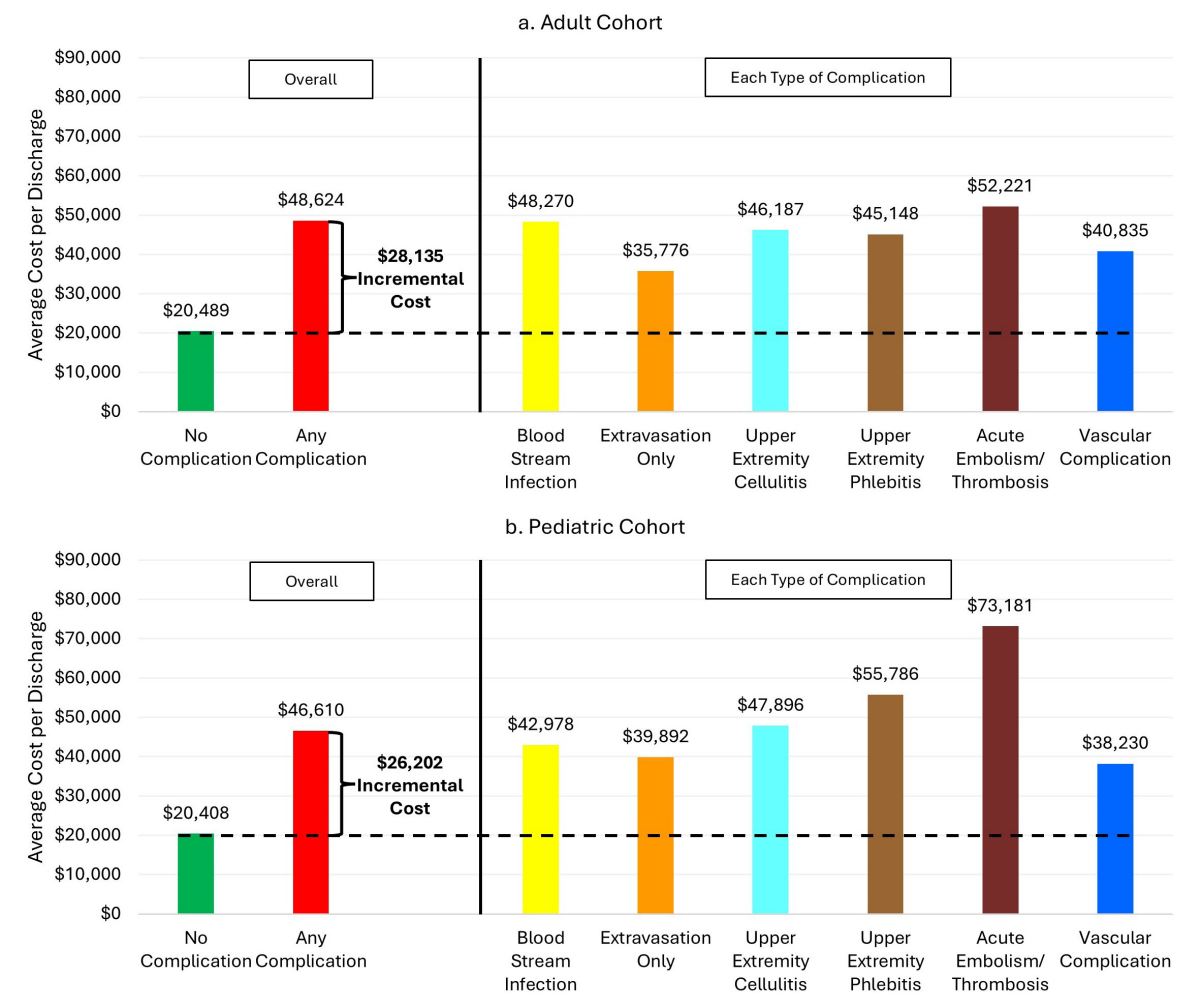
Hospitalization Cost Between Surgical Inpatients with and without Peripheral Intravenous Catheter–Associated Complications, for Adult (**a**) and Pediatric (**b**) Cohorts

In the adult cohort, the mean total hospitalization cost was substantially higher among patients with PIVC-associated complications compared to those without ($48 624 vs $20 489, *P* < .01) (**Tables 2 and 4**). The total hospitalization cost for each type of PIVC-associated complications was higher than that of patients without complications, and the cost was the highest for patients with acute embolism/thrombosis (**Figure 1a**). Although the difference attenuated after adjusting for covariates, patients with PIVC-associated complications incurred 93% higher cost (adjusted cost ratio, 1.93; 95% CI, 1.92-1.94) than those without complications, amounting to the adjusted mean difference of $19 074.

Similarly in the pediatric cohort, the mean total hospitalization cost was significantly higher among patients with PIVC-associated complications compared to those without ($46 610 vs $20 407, *P* < .01) (**Tables 2 and 4**). As with the adult cohort, the total hospitalization cost was the highest for pediatric patients with acute embolism/thrombosis (**Figure 1b**). Following adjustment for covariates, patients with PIVC-associated complications incurred 78% higher cost (adjusted cost ratio, 1.78; 95% CI, 1.67-1.89) than those without, amounting to the adjusted mean difference of $16 052.

## DISCUSSION

This large, retrospective observational analysis of hospitalized surgical patients indicates evidence of PIVC-associated complications and their healthcare burden. While the overall prevalence of PIVC-associated complications may seem low at around 1%, this still amounts to a significant number of patients (eg, ~52 000 patients just in this study), as most patients undergoing an inpatient surgical procedure would have a PIVC placed. The results from this study indicated that patients with PIVC-associated complications were more likely to be hospitalized longer, readmitted within 30 days, and incur higher hospitalization cost than patients without these complications. This observation was consistent for both adult and pediatric patients, and before and after adjusting for potential confounders.

Lim et al^30^ reported that about 2% of 588 375 hospitalized patients with pneumonia, chronic obstructive pulmonary disease, myocardial infarction, congestive heart failure, chronic kidney disease, diabetes with complications, and major trauma developed PIVC-associated complications. Among these various conditions, patients with pneumonia, diabetes, or myocardial infarction had the highest prevalence of complications. The prevalence of PIVC-associated complications was lower (~1%) in our study, which is plausible as our study population would have been relatively healthier to undergo a surgical procedure compared to the population who had been hospitalized for infections and chronic conditions. Almost half of our adult study population had no chronic conditions as evidenced by Charlson-Deyo Comorbidity Index score of zero. This is also similar to reporting of Lim et al^30^ that the prevalence of PIVC-associated complications among major trauma patients was 1.03%.

We found that among different types of PIVC-associated complications, BSI was most prevalent (0.52% in the adult cohort and 0.37% in the pediatric cohort), which is consistent with the findings by Lim et al.^30^ Prevalence of PIVC-associated BSI in this study is higher than that in a systemic review and meta-analysis by Marsh et al^1^ (1 in 5606). However, as the authors stated, PIVC-associated BSIs often do not present signs and symptoms, necessitating prompt tip cultures that leads to accurate diagnosis. This most likely resulted in underestimations for clinical studies included in the meta-analysis (as opposed to this study and the Lim et al study using real-world data).^30^ Even at a rate of 1 in 5606 (~0.02%) for PIVC-associated BSI, Marsh et al^1^ argued that it is comparable to the rate of PICC- and CVC-related BSIs, given the enormous volume of PIVC use.

We note that a systemic review of complications associated with with long peripheral catheters showed that the most common complications were catheter occlusion (4%), followed by phlebitis (1%), infiltration (0.9%), and catheter-associated BSI (0.3%).^3^ Because our study used a real-world hospital administrative database, it is possible that complications such as catheter occlusion and infiltration, which are often perceived as less severe but potentially clinically significant, were underreported.

While discrepancies exist in the magnitude of healthcare burden across different studies, this study and others suggest that PIVC-associated complications are costly and a significant burden to hospitals.

Studies have called to action on reducing PIVC-associated complications through a multistage approach: by identifying it as a public health problem, developing and implementing quality measures and best practices for the insertion of PIVC, and creating federal and state prevention programs similarly to the successful approaches taken for central line-associated BSIs.^1,30–32^

We want to add that establishing a formal mechanism to better track the incidence of PIVC-associated BSIs, thus leading to robust prevention programs and enhanced care, could be a practical investment for all stakeholders in the US healthcare system.

This study is not without limitations. First, the use of PIVC was inferred rather than directly observed. Because the use of PIVC is difficult to capture in real-world data, we assumed that all inpatient surgical procedures require vascular access for administering anesthesia. By excluding patients with PICCs or CVCs, we further assumed that all remaining patients had a PIVC in place. Based on the literature and clinical expert input, the placement of a PIVC in every patient upon admission is a standard practice.^30^ Because we expected all patients included in the study to have a PIVC placed, misclassification of PIVC use is possible if some surgical patients did not receive a PIVC. Second, complications were identified using ICD-10 codes and may have undercaptured less severe but clinically relevant events (eg, occlusion or infiltration) and may have been subject to variability in coding practices across hospitals. The resulting association due to misclassification of undercaptured events is likely biased toward the null. Third, the timing of complications relative to outcomes occurring during the hospitalization (ie, LOS and total hospitalization cost) could not be determined and included days and costs prior to the complication, limiting causal attribution. As with the nature of real-world data study, because the date of complication is difficult to determine without the presence of clinical notes and because patients without complications would not have reference dates, it was not possible to capture outcomes only happening after the complication during hospitalization. By adjusting for appropriate baseline conditions and confounders, the difference in outcomes is expected to be the effect of PIVC-associated complications. Fourth, readmissions and outpatient visits to the same hospital or health system are captured, but visits to a different PHD hospital during the follow-up period may have been missed. Because the observation period is short for this study, attrition due to relocation is not expected, and patients were expected to return to the same hospital if they experienced any health issues after discharge. Therefore, the frequency of missed visits to a different hospital is expected to be very low. Lastly, residual confounding is possible, as important factors such as catheter dwell time, number of insertion attempts, and provider skill were not captured and because variable selection was based on statistical significance. However, a priori variable selection was based on clinical relevance, and retaining all covariates regardless of *P* value did not change the interpretation of the results.

## CONCLUSIONS

This study indicated that PIVC-associated complications were associated with increased cost, extended hospital length of stay and increased risk of readmission during 30-day follow-up in both adult and pediatric surgical inpatients in the United States. This is one of the first few studies to report on the real-world costs to hospitals associated with PIVC-associated complications among inpatients undergoing surgeries. The results of this study can serve as a catalyst for stakeholders to establish a process for decreasing complications related to the use of PIVCs. A comprehensive understanding of the implications to patients, healthcare providers, and payers requires an investment in detailed research to quantify the magnitude of the problem, explore causality, and most importantly, determine those complications that are potentially avoidable when considered with fiscally responsible solutions.

### Disclosures

S.P. and K.J. are employees of B. Braun Medical Inc. B. Braun Medical Inc. contracted Premier, Inc. to conduct this study. J.G., R.M., J.D., and N.R. worked on this study as employees of Premier Inc.

## Supplementary Material

Online Supplementary Material
