# Supplementary material for: Peripheral Intravenous Catheters in Hospitalized Surgical Patients: A US Real-World Data Analysis
Source: J Health Econ Outcomes Res. 2026 Apr 10;13(1):120–9. doi: 10.36469/001c.156489 (PMC13070356; doi:10.36469/001c.156489)
Supplement: Online Supplementary Material [file jheor_2026_13_1_156489_339406.pdf]

Journal of Health Economics and Outcomes Research

Online Supplementary Material

Peripheral Intravenous Catheters in Hospitalized Surgical Patients: A US Real-World Data Analysis. *JHEOR*. 2026;13(1):120-129. doi:10.36469/jheor.2026.156489

Table S1: ICD-10 Diagnosis Codes for Complications

Table S2A: Patient Demographics, Clinical Characteristics, and Hospital Characteristics of Adult Surgical Inpatients with PIVC-Associated Complications

Table S2B: Patient Demographics, Clinical Characteristics, and Hospital Characteristics of Pediatric Surgical Inpatients with PIVC-Associated Complications

Figure S1: Patient Selection Flowchart

This supplementary material has been provided by the authors to give readers additional information about their work.

This is an open-access article distributed under the terms of the Creative Commons Attribution 4.0 International License (CCBY-4.0). View this license's legal deed at http://creativecommons.org/licenses/by/4.0 and legal code at http://creativecommons.org/licenses/by/4.0/legalcode for more information.

Table S1. ICD-10 Diagnosis Codes for Complications

Subcategory Description	ICD-10-CM Codes	ICD-10-CM Description
Bloodstream infection	A02.1	*Salmonella* sepsis
	A22.7	Anthrax sepsis
	A26.7	*Erysipelothrix* sepsis
	A32.7	Listerial sepsis
	A40.%	Streptococcal sepsis
	A41.0%	Sepsis due to *Staphylococcus aureus*
	A41.1	Sepsis due to other specified staphylococcus
	A41.2	Sepsis due to unspecified staphylococcus
	A41.3	Sepsis due to *Hemophilus influenzae*
	A41.4	Sepsis due to anaerobes
	A41.50	Gram-negative sepsis, unspecified
	A41.51	Sepsis due to *Escherichia coli* [*E. coli*]
	A41.52	Sepsis due to *Pseudomonas*
	A41.53	Sepsis due to *Serratia*
	A41.59	Other gram-negative sepsis
	A41.81	Sepsis due to *Enterococcus*
	A41.89	Other specified sepsis
	A41.9	Sepsis, unspecified organism
	A42.7	Actinomycotic sepsis
	A54.86	Gonococcal sepsis
	B37.7	Candidal sepsis
	R65.2%	Severe sepsis
	R65.20	Severe sepsis without septic shock
	R65.21	Severe sepsis with septic shock
	R78.81	Bacteremia
	T81.44%	Sepsis following a procedure
Extravasation	T80.81%	Extravasation (including infiltration) of vesicant agent
	T80.89XA	Other complications following infusion, transfusion and therapeutic injection, initial encounter
	T80.89XD	Other complications following infusion, transfusion and therapeutic injection, subsequent encounter
	T80.89XS	Other complications following infusion, transfusion and therapeutic injection, sequela
	T80.90%	Unspecified complication following infusion and therapeutic injection
Upper extremity cellulitis	L03.113	Cellulitis of right upper limb
	L03.114	Cellulitis of left upper limb
	L03.123	Acute lymphangitis of right upper limb
	L03.124	Acute lymphangitis of left upper limb
Upper extremity phlebitis	I80.8	Phlebitis and thrombophlebitis of other sites
	I80.9	Phlebitis and thrombophlebitis of unspecified site
Acute embolism/thrombosis	I82.90	Acute embolism and thrombosis of unspecified vein
Vascular complications	T80.1XXA	Vascular complications following infusion, transfusion and therapeutic injection, initial encounter
	T80.1XXD	Vascular complications following infusion, transfusion and therapeutic injection, subsequent encounter
	T80.1XXS	Vascular complications following infusion, transfusion and therapeutic injection, sequela
	T81.72%	Complication of vein following a procedure, not elsewhere classified

Table S2A. Patient Demographics, Clinical Characteristics, and Hospital Characteristics of Adult Surgical Inpatients with PIVC-Associated Complications

**Characteristics**	**Overall (N = 6 992 120)**		**Patients with PIVC-Associated Complications (N = 51 294)**		**Patients without PIVC-Associated Complications (N = 6 940 826)**		***P***
	**n**	**%**	**n**	**%**	**n**	**%**	
**Patient demographics**							
Age, y[a]	55.3 (20)		64.2 (17)		55.2 (20)		<.0001
Age category, n (%)							
18-34 y	1 576 129	22.7	4106	8.1	1 572 023	22.9	<.0001
35-49 y	1 165 346	16.8	5882	11.6	1 159 464	16.9	
50-64 y	1 508 068	21.8	12 880	25.5	1 495 188	21.7	
65-74 y	1 366 660	19.7	12 461	24.7	1 354 199	19.7	
75-84 y	966 836	14.0	10 673	21.1	956 163	13.9	
85+ y	347 261	5.0	4549	9.0	342 712	5.0	
Gender, n (%)							
Male	2 569 108	36.8	27 239	53.1	2 541 869	36.6	<.0001
Female	4 421 303	63.2	24 046	46.9	4 397 257	63.4	
Race, n (%)							
White	5 096 804	72.9	35 554	69.3	5 061 250	72.9	<.0001
Black	904 883	12.9	8241	16.1	896 642	12.9	
Other	990 433	14.2	7499	14.6	982 934	14.2	
Ethnicity, n (%)							
Hispanic	825 112	11.8	5525	10.8	819 587	11.8	<.0001
Non-Hispanic	5 325 717	76.2	38 597	75.2	5 287 120	76.2	
Unknown	841 291	12.0	7172	14.0	834 119	12.0	
Health insurance type, n (%)							
Medicare	2 844 190	40.7	29 814	58.1	2 814 376	40.5	<.0001
Medicaid	1 246 771	17.8	8023	15.6	1 238 748	17.8	
Private insurance	2 390 314	34.2	10 205	19.9	2 380 109	34.3	
Uninsured	226 795	3.2	1531	3.0	225 264	3.2	
Other/unknown	284 050	4.1	1721	3.4	282 329	4.1	
Admission point of origin, n (%)							
Clinic	1 239 763	17.7	4641	9.0	1 235 122	17.8	<.0001
Non-healthcare (eg, home)	5 221 372	74.7	37 634	73.4	5 183 738	74.7	
Transfer from an acute care hospital	420 984	6.0	7428	14.5	413 556	6.0	
Transfer from long-term care or rehab facility	46 880	0.7	1124	2.2	45 756	0.7	
Other/unknown	63 121	0.9	467	0.9	62 654	0.9	
Admission type, n (%)							
Elective	3 427 370	49.0	8855	17.3	3 418 515	49.3	<.0001
Emergency	2 409 261	34.5	32 045	62.5	2 377 216	34.2	
Urgent	920 155	13.2	8323	16.2	911 832	13.1	
Trauma or injury center	134 465	1.9	1654	3.2	132 811	1.9	
Other unknown	100 869	1.4	417	0.8	100 452	1.4	
Discharge status, n (%)							
Home/home health	5 894 423	84.3	24 188	47.2	5 870 235	84.6	<.0001
Nursing or rehabilitation facility	915 652	13.1	17 012	33.2	898 640	12.9	

Table S2A. Patient Demographics, Clinical Characteristics, and Hospital Characteristics of Adult Surgical Inpatients with PIVC-Associated Complications

Characteristics	Overall (N = 6 992 120)		Patients with PIVC-Associated Complications (N = 51 294)		Patients without PIVC-Associated Complications (N = 6 940 826)		*P*
	n	%	n	%	n	%	
Transferred to an acute care hospital	18 420	0.3	327	0.6	18 093	0.3	<.0001
Hospice	39 029	0.6	2574	5.0	36 455	0.5	
Expired	37 895	0.5	5558	10.8	32 337	0.5	
Other/unknown	86 701	1.2	1635	3.2	85 066	1.2	
**Hospital characteristics**							
Hospital setting, n (%)							
Urban	6 400 846	91.5	47 403	92.4	6 353 443	91.5	<.0001
Rural	591 274	8.5	3891	7.6	587 383	8.5	
Teaching status, n (%)							
Teaching	3 927 730	56.2	31 335	61.1	3 896 395	56.1	<.0001
Nonteaching	3 064 390	43.8	19 959	38.9	3 044 431	43.9	
Geographic region, n (%)							
Midwest	1 467 271	21.0	9971	19.4	1 457 300	21.0	<.0001
Northeast	1 290 862	18.5	11 383	22.2	1 279 479	18.4	
South	3 205 069	45.8	22 278	43.4	3 182 791	45.9	
West	1 028 918	14.7	7662	14.9	1 021 256	14.7	
Hospital size, n (%)							
<100 beds	340 968	4.9	1704	3.3	339 264	4.9	<.0001
100-199 beds	934 502	13.4	5602	10.9	928 900	13.4	
200-299 beds	1 104 120	15.8	7826	15.3	1 096 294	15.8	
300-499 beds	1 920 658	27.5	13 619	26.6	1 907 039	27.5	
500+ beds	2 685 721	38.4	22 451	43.8	2 663 270	38.4	
Unknown	6151	0.1	92	0.2	6 059	0.1	
**Clinical Characteristics**							
Charlson-Deyo Comorbidities, n (%)							
Myocardial infarction	359 453	5.1	5745	11.2	353 708	5.1	<.0001
Congestive heart failure	663 037	9.5	12 985	25.3	650 052	9.4	<.0001
Peripheral vascular disease	299 420	4.3	4793	9.3	294 627	4.2	<.0001
Cerebrovascular disease	324 590	4.6	6626	12.9	317 964	4.6	<.0001
Dementia	241 240	3.5	4598	9.0	236 642	3.4	<.0001
Chronic pulmonary disease	1 165 700	16.7	13 748	26.8	1 151 952	16.6	<.0001
Rheumatic disease	143 958	2.1	1478	2.9	142 480	2.1	<.0001
Peptic ulcer disease	50 877	0.7	1881	3.7	48 996	0.7	<.0001
Mild liver disease	202 156	2.9	2467	4.8	199 689	2.9	<.0001
Diabetes without chronic complication	796 311	11.4	7089	13.8	789 222	11.4	<.0001
Diabetes with chronic complication	719 147	10.3	11 134	21.7	708 013	10.2	<.0001
Hemiplegia or paraplegia	94 926	1.4	3006	5.9	91 920	1.3	<.0001
Renal disease	1 128 249	16.1	25 244	49.2	1 103 005	15.9	<.0001
Moderate/severe liver disease	73 537	1.1	2894	5.6	70 643	1.0	<.0001
Any malignancy	637 016	9.1	8923	17.4	628 093	9.0	<.0001

Table S2A. Patient Demographics, Clinical Characteristics, and Hospital Characteristics of Adult Surgical Inpatients with PIVC-Associated Complications

Characteristics	Overall (N = 6 992 120)		Patients with PIVC-Associated Complications (N = 51 294)		Patients without PIVC-Associated Complications (N = 6 940 826)		*P*
	n	%	n	%	n	%	
Metastatic solid tumor	199 547	2.9	4054	7.9	195 493	2.8	<.0001
HIV disease	9653	0.1	219	0.4	9434	0.1	<.0001
Charlson-Deyo Comorbidity Index (CCI) score category							
0	3 283 879	47.0	7649	14.9	3 276 230	47.2	<.0001
1-2	2 097 890	30.0	12 838	25.0	2 085 052	30.0	
>2	1 610 351	23.0	30 807	60.1	1 579 544	22.8	
CCI score[a]	1.56 (2)		3.75 (3)		1.54 (2)		<.0001

Abbreviation: PIVC, peripheral intravenous catheter.
[a]Mean (SD).

Table S2B. Patient Demographics, Clinical Characteristics, and Hospital Characteristics of Pediatric Surgical Inpatients with PIVC-Associated Complications

Characteristics	Overall (N = 159 256)		Patients with PIVC-Associated Complications (N = 816)		Patients without PIVC-Associated Complications (N = 158 440)		*P*
	n	%	n	%	n	%	
**Patient demographics**							
Age, y[a]	9.5	6	8.6	6	9.6	6	<.0001
Age category, n (%)							
<1 y	26 749	16.8	168	20.6	26 581	16.8	.0084
2-5 y	21 447	13.5	115	14.1	21 332	13.5	
6-17 y	111 060	69.7	533	65.3	110 527	69.8	
Gender, n (%)							
Male	85 610	53.8	433	53.1	85 177	53.8	.6867
Female	73 617	46.2	383	46.9	73 234	46.2	
Race, n (%)							
White	95 889	60.2	485	59.4	95 404	60.2	.0011
Black	24 862	15.6	162	19.9	24 700	15.6	
Other	38 505	24.2	169	20.7	38 336	24.2	
Ethnicity, n (%)							
Hispanic	38 721	24.3	167	20.5	38 554	24.3	<.0001
Non-Hispanic	101 121	63.5	401	49.1	100 720	63.6	
Unknown	19 414	12.2	248	30.4	19 166	12.1	
Health insurance type, n (%)							
Medicare	235	0.1	3	0.4	232	0.1	<.0001
Medicaid	88 446	55.5	497	60.9	87 949	55.5	
Private insurance	60 161	37.8	262	32.1	59 899	37.8	
Uninsured	3791	2.4	35	4.3	3 756	2.4	
Other/unknown	6623	4.2	19	2.3	6604	4.2	
Admission point of origin, n (%)							
Clinic	22 228	14.0	88	10.8	22 140	14.0	.029
Non-healthcare (eg, home)	113 813	71.5	590	72.3	113 223	71.5	
Transfer from an acute care hospital	21 915	13.8	127	15.6	21 788	13.8	

Table S2B. Patient Demographics, Clinical Characteristics, and Hospital Characteristics of Pediatric Surgical Inpatients with PIVC-Associated Complications

**Characteristics**	**Overall (N = 159 256)**		**Patients with PIVC-Associated Complications (N = 816)**		**Patients without PIVC-Associated Complications (N = 158 440)**		***P***
	**n**	**%**	**n**	**%**	**n**	**%**	
Transfer from long-term care or rehab facility	107	0.1	1	0.1	106	0.1	.029
Other/unknown	1193	0.7	10	1.2	1 183	0.7	
Admission type, n (%)							
Elective	53 635	33.7	161	19.7	53 474	33.8	<.0001
Emergency	70 018	44.0	464	56.9	69 554	43.9	
Urgent	26 011	16.3	129	15.8	25 882	16.3	
Trauma or injury center	8276	5.2	52	6.4	8224	5.2	
Other/unknown	1316	0.8	10	1.2	1306	0.8	
Discharge status, n (%)							
Home/home health	155 376	97.6	706	86.5	154 670	97.6	<.0001
Nursing or rehabilitation facility	1663	1.0	47	5.8	1616	1.0	
Transferred to an acute care hospital	429	0.3	11	1.3	418	0.3	
Hospice	51	0.0	2	0.2	49	0.0	
Expired	317	0.2	22	2.7	295	0.2	
Other/unknown	1420	0.9	28	3.4	1392	0.9	
**Hospital characteristics**							
Hospital setting, n (%)							
Urban	153 585	96.4	802	98.3	152 783	96.4	.0043
Rural	5671	3.6	14	1.7	5657	3.6	
Teaching status, n (%)							
Teaching	122 367	76.8	696	85.3	121 671	76.8	<.0001
Nonteaching	36 889	23.2	120	14.7	36 769	23.2	
Geographic region, n (%)							
Midwest	22 848	14.3	86	10.5	22 762	14.4	<.0001
Northeast	31 233	19.6	102	12.5	31 131	19.6	
South	83 057	52.2	571	70.0	82 486	52.1	
West	22 118	13.9	57	7.0	22 061	13.9	
Hospital size, n (%)							
<100 beds	3688	2.3	6	0.7	3682	2.3	<.0001
100-199 beds	26 995	17.0	67	8.2	26 928	17.0	
200-299 beds	20 934	13.1	327	40.1	20 607	13.0	
300-499 beds	25 239	15.8	95	11.6	25 144	15.9	
500+ beds	82 388	51.7	321	39.3	82 067	51.8	
Unknown	12	0.0	0	0.0	12	0.0	
**Clinical characteristics**							
Feudtner Complex Chronic Conditions, n (%)							
Neurologic and neuromuscular	14 874	9.3	119	14.6	14 755	9.3	<.0001
Cardiovascular	13 334	8.4	163	20.0	13 171	8.3	<.0001
Respiratory	4870	3.1	88	10.8	4782	3.0	<.0001
Renal and urologic	5664	3.6	52	6.4	5612	3.5	<.0001
Gastrointestinal	12 930	8.1	175	21.4	12 755	8.1	<.0001

Table S2B. Patient Demographics, Clinical Characteristics, and Hospital Characteristics of Pediatric Surgical Inpatients with PIVC-Associated Complications

**Characteristics**	**Overall (N = 159 256)**		**Patients with PIVC-Associated Complications (N = 816)**		**Patients without PIVC-Associated Complications (N = 158 440)**		***P***
	**n**	**%**	**n**	**%**	**n**	**%**	
Hematologic or immunologic	3781	2.4	73	8.9	3708	2.3	<.0001
Metabolic	5138	3.2	106	13.0	5032	3.2	<.0001
Other congenital or genetic defect	0	0.0	0	0.0	0	0.0	NA
Premature and neonatal	1817	1.1	51	6.2	1766	1.1	<.0001
Malignancy	4670	2.9	45	5.5	4625	2.9	<.0001
Technology dependence	14 308	9.0	185	22.7	14 123	8.9	<.0001
Solid organ/bone marrow transplantation	609	0.4	13	1.6	596	0.4	<.0001

Abbreviations: NA, not applicable; PIVC, peripheral intravenous catheter.
[a]Mean (SD).

Figure S1. Patient Selection Flowchart

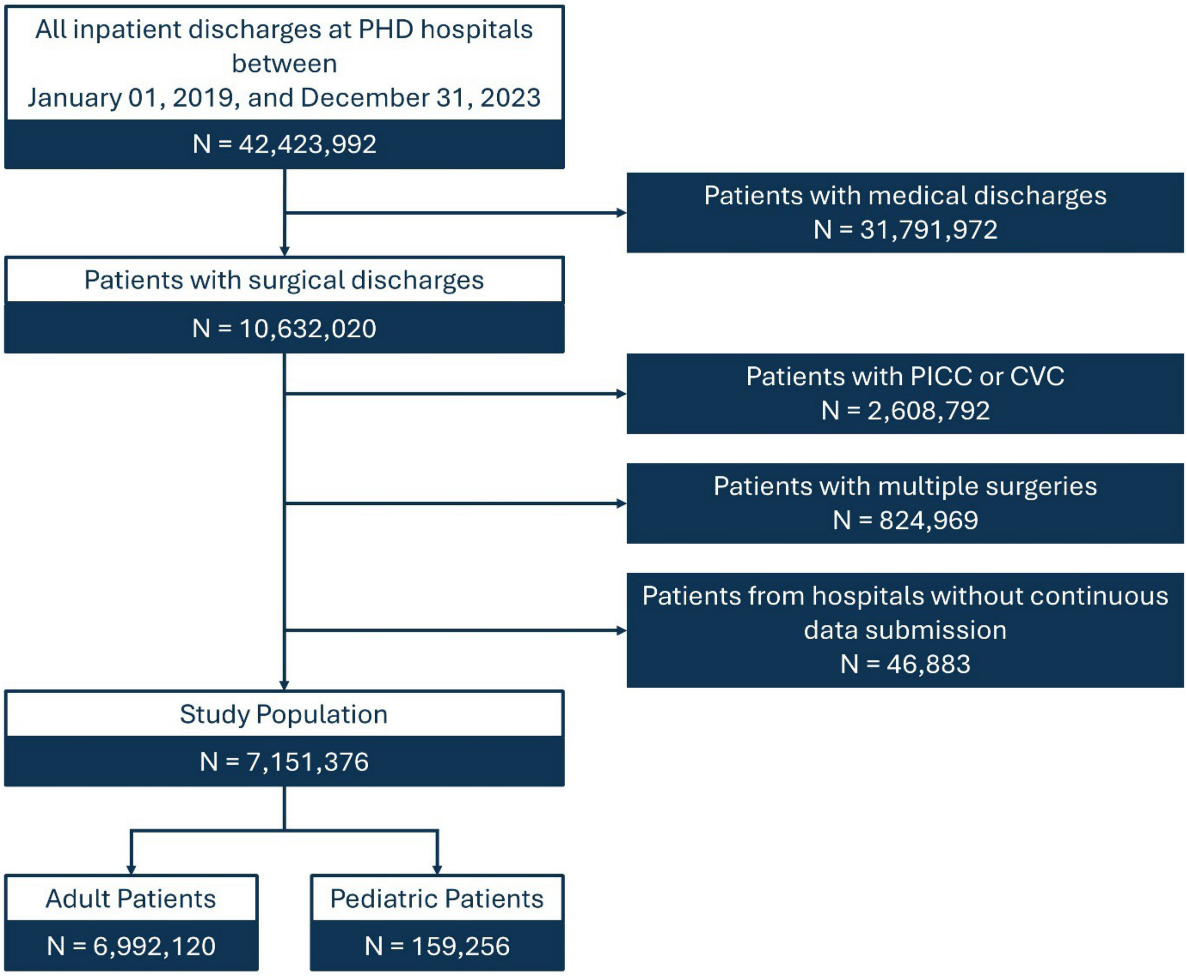

Abbreviations: CVC, central venous catheter; PHD, Premier Healthcare Database: PICC, peripherally inserted central catheter.
